# Endothelial cell activation, Weibel-Palade body secretion, and enhanced angiogenesis in severe COVID-19

**DOI:** 10.1016/j.rpth.2023.100085

**Published:** 2023-02-14

**Authors:** Ellie Karampini, Helen Fogarty, Stephanie Elliott, Hannah Morrin, Colm Bergin, Jamie M. O’Sullivan, Mary Byrne, Ignacio Martin-Loeches, Patrick W. Mallon, Gerard F. Curley, Siobhan Glavey, Ross I. Baker, M. Lavin, Roger J.S. Preston, Cliona Ni Cheallaigh, Soracha E. Ward, James S. O’Donnell

**Affiliations:** 1 Irish Centre for Vascular Biology, School of Pharmacy and Biomolecular Sciences, Royal College of Surgeons in Ireland, Dublin, Ireland; 2 Department of Infectious Diseases, St James’s Hospital, Dublin, Ireland; 3 Department of Clinical Medicine, School of Medicine, Trinity Translational Medicine Institute, Trinity College Dublin, Dublin, Ireland; 4 National Coagulation Centre, St James’s Hospital, Dublin, Ireland; 5 Department of Intensive Care Medicine, St James’s Hospital, Dublin, Ireland; 6 Centre for Experimental Pathogen Host Research, University College Dublin, Dublin, Ireland; 7 St Vincent’s University Hospital, Dublin, Ireland; 8 Department of Anaesthesia and Critical Care, Royal College of Surgeons in Ireland, Dublin, Ireland; 9 Department of Haematology, Beaumont Hospital, Dublin, Ireland; 10 Royal College of Surgeons in Ireland, Dublin, Ireland; 11 Western Australia Centre for Thrombosis and Haemostasis, Perth Blood Institute, Murdoch University, Perth, Western Australia, Australia; 12 Irish-Australian Blood Collaborative Network, Dublin, Ireland; 13 National Children’s Research Centre, Our Lady’s Children’s Hospital Crumlin, Dublin, Ireland

**Keywords:** angiogenesis, COVID-19, endothelial activation, endothelial cells, Weibel-Palade body

## Abstract

**Background:**

Severe COVID-19 is associated with marked endothelial cell (EC) activation that plays a key role in immunothrombosis and pulmonary microvascular occlusion. However, the biological mechanisms through which SARS-CoV-2 causes EC activation and damage remain poorly defined.

**Objectives:**

We investigated EC activation in patients with acute COVID-19, and specifically focused on how proteins stored within Weibel-Palade bodies may impact key aspects of disease pathogenesis.

**Methods:**

Thirty-nine patients with confirmed COVID-19 were recruited. Weibel-Palade body biomarkers (von Willebrand factor [VWF], angiopoietin-2 [Angpt-2], and osteoprotegerin) and soluble thrombomodulin (sTM) levels were determined. In addition, EC activation and angiogenesis were assessed in the presence or absence of COVID-19 plasma incubation.

**Results:**

Markedly elevated plasma VWF antigen, Angpt-2, osteoprotegerin, and sTM levels were observed in patients with acute COVID-19. The increased levels of both sTM and Weibel-Palade body components (VWF, osteoprotegerin, and Angpt-2) correlated with COVID-19 severity. Incubation of COVID-19 plasma with ECs triggered enhanced VWF secretion and increased Angpt-2 expression, as well as significantly enhanced *in vitro* EC tube formation and angiogenesis.

**Conclusion:**

We propose that acute SARS-CoV-2 infection leads to a complex and multifactorial EC activation, progressive loss of thrombomodulin, and increased Angpt-2 expression, which collectively serve to promote a local proangiogenic state.

## Essentials

• Severe COVID-19 is associated with endotheliopathy that plays a key role in immunothrombosis.
• Endothelial cell activation correlates with COVID-19 severity and results in Weibel-Palade body exocytosis.
• Plasma levels of von Willebrand factor, angiopoietin-2, osteoprotegerin, and soluble thrombomodulin are elevated in patients with severe COVID-19.
• Plasma alterations result in endothelial cell activation and Weibel-Palade body secretion and may promote local angiogenesis.

## 1 Introduction

SARS-CoV-2 is responsible for the COVID-19 pandemic that has already resulted in >200 million cases and >5 million deaths worldwide. Mortality is primarily due to severe bilateral pneumonitis, resulting in acute respiratory distress syndrome. Postmortem studies on COVID-19 have demonstrated the presence of fibrin-rich microthrombi disseminated throughout the pulmonary microvasculature. [[1], [2], [3]] Under normal conditions, endothelial cells (EC) function to prevent thrombosis. In patients with fatal COVID-19, evidence of significant endotheliopathy has also been observed at autopsy. [4] Its features include EC swelling, disruption of normal tight EC junctions with enhanced-barrier permeability, and enhanced EC apoptosis. [5] Significantly increased intussusceptive angiogenesis within the lungs is another hallmark of severe COVID-19.

In keeping with the postmortem findings, biomarkers of EC activation are significantly elevated in acute COVID-19. [6] For example, plasma von Willebrand factor antigen (VWF:Ag) and VWF propeptide levels are increased by ∼8- to 10-fold in patients with COVID-19 requiring intensive care unit (ICU) admission. [7,8] VWF:Ag and VWF propeptide levels also correlate with COVID-19 severity. Furthermore, reduced ADAMTS-13 levels and ultralarge VWF multimers have been observed in severe COVID-19. [9] Collectively, these observations suggest that dysfunction of the VWF-ADAMTS-13 axis plays a role in COVID-19 pulmonary microvascular occlusion. Under steady-state conditions, ultralarge VWF multimers are stored in Weibel-Palade bodies (WPBs) within ECs. Importantly, WPBs also store a number of other proinflammatory and proangiogenic molecules, including angiopoietin-2 (Angpt-2) and osteoprotegerin (OPG). [10] Acute EC activation triggers WPB exocytosis and secretion of these glycoproteins into the vessel lumen, where they impact local hemostasis, inflammation, and angiogenesis. [11]

Although endotheliopathy has been implicated as playing a key role in COVID-19 pathogenesis, the biological mechanisms through which SARS-CoV-2 causes EC activation and damage are not well defined. Early electron microscopy studies reported the presence of SARS-CoV-2 within EC. [12] However, more recent studies suggest that ECs are actually resistant to direct infection. [13] In this study, we investigated EC activation in patients with acute COVID-19, and specifically focused on how proteins stored within WPB may impact key aspects of disease pathogenesis.

## 2 Methods

### 2.1 Patients

Consecutive adult patients (aged >18 years) with acute COVID-19 (confirmed by a positive SARS-CoV-2 polymerase chain reaction test) admitted to St James’s and Beaumont Hospitals, Dublin, between March 21 and May 6, 2020, were eligible for enrolment. Children and pregnant or breastfeeding women were excluded from the study. A control group of asymptomatic healthy controls (n = 15) was also recruited. The study was approved by the local hospital research ethics committees (references REC 2020-03 and REC 17/06). Written informed consent was obtained from all the patients. For unconscious patients who were intubated and ventilated in the ICU, assent to participate in the study was obtained initially from the next of kin if patients lacked capacity and informed consent was retrospectively obtained wherever possible from participants once capacity was regained. All participants received standard dose low-molecular-weight heparin thromboprophylaxis according to weight and renal function as previously described. [7] Patients with COVID-19 treated at the ward level were classified as “moderate,” whereas patients requiring ICU admission were classified as “critical.” Regarding respiratory support, moderate cases required supplemental oxygen (median FiO_2_ requirement: 54%), whereas critical patients required intubation and ventilation. Patients who died from COVID-19–related complications were classified as “fatal” (Table). Blood samples were collected in 3.2% sodium citrate tubes. Plasma VWF:Ag levels were determined by standard ELISA as before. [7] Plasma angiopoietin-2, osteoprotegerin, and soluble thrombomodulin (sTM) levels were measured using commercial ELISAs performed according to the manufacturers’ instructions.

**Table:** Demographic, clinical, and laboratory characteristics of patients with COVID-19.

Characteristics	Normal range	Moderate (n = 14)	Critical (n = 16)	Fatal (n = 9)
Age, y (mean ± SD)		69 ± 14	54 ± 11	68 ± 14
Male–*n* (%)		7 (50)	14 (88)	8 (89)
Caucasian–*n* (%)		11 (79)	8 (50)	6 (66.7)
Asian–*n* (%)		2 (14)	6 (37.5)	2 (22.2)
Romanian–*n* (%)		0 (0)	2 (12.5)	1 (11.1)
African–*n* (%)		1 (7)	0 (0)	0 (0)
Body mass index (BMI), kg/m^2^ (mean ± SD)		25 ± 7.6	33 ± 7.5	30.1 ± 5.2
**Medical comorbidities–*n* (%)**				
Obesity (BMI > 30 kg/m^2^)		2 (14)	11 (69)	5 (56)
Hypertension		7 (50)	9 (56)	4 (44)
Chronic respiratory disease		2 (14)	4 (25)	3 (33)
Hyperlipidemia		4 (29)	5 (32)	2 (22)
Diabetes		4 (29)	3 (19)	2 (22)
Cardiovascular disease		6 (43)	2 (13)	5 (56)
Chronic kidney disease		2 (14)	2 (13)	1 (11)
**Baseline laboratory parameters median (IQR) unless otherwise stated**				
Leukocytes (× 10^9^/L)	4-11	5.7 (2.5)	11.5 (5.3)	7.3 (0.5)
Neutrophils (× 10^9^/L)	2-7.5	3.35 (2.2)	10.3 (4)	5.9 (0.4)
Lymphocytes (× 10^9^/L)	1.5-3.5	1.4 (0.8)	0.9 (0.7)	0.9 (0.1)
Platelets (× 10^9^/L)	140-450	295 (121.5)	268 (150)	282 (155)
PT (s)	9.9-13.1	12.1 (1.3)	13.9 (2.1)	15.4 (2.7)
aPTT (s) mean ± SD	24-36	32 ± 4.7	30.1 ± 4.2	28.6 ± 7.0
Ferritin (ng/mL)	23-393	540 (533)	1938 (1074)	1143 (223)
Fibrinogen (g/L)	1.9-3.5	4.4 (2.3)	6.5 (3)	4.8 (0.75)
D-dimer (ng/mL)	0-500	879.5 (755)	1005 (1393)	6965 (6126)
Creatinine (μmol/L)	45-84	68 (28)	77.5 (19.3)	95 (26)
CRP (mg/L)	0-5	23 (62.2)	154 (210)	199 (48)
Il-6 (pg/mL)	0.09-7.26	12.2 (15)	93.9 (87.3)	213.8 (87.3)
ISTH DIC Score	<5	2 (0)	2 (0)	3 (1)
**Clinical outcome measures**				
Invasive mechanical ventilation–*n* (%)		0 (0)	16 (100)	4 (44)
Length of ICU stay (d)–median (range)		NA	25 (6-79)	0 (0-36)
Length of hospital stay (d)–median (range)		16.5 (4-116)	43 (13-125)	20 (7-66)
Pulmonary thrombosis–*n* (%)		3 (21)	4 (25)	3 (33)
In-hospital death–*n* (%)		0 (0)	0 (0)	9 (100)

aPTT, activated partial thromboplastin time; CRP, C-reactive protein; ICU, intensive care unit; IL, interleukin; ISTH DIC, ISTH criteria for Disseminated Intravascular Coagulation; PT, prothrombin time.

### 2.2 Endothelial cell treatment with plasma

To investigate the effect of COVID-19 plasma on ECs, human umbilical vein ECs (HUVECs) were treated with control or patient plasma (diluted 1:2 in release medium [RM: Medium M199 supplemented with 0.2% bovine serum albumin]) for 10 minutes (37 °C, 5% CO_2_). After washing (×3 with sterile phosphate-buffered saline) to remove the plasma, HUVECs were incubated in RM for 30 minutes. Supernatants were collected and secreted VWF:Ag levels were determined. Values from control and patient plasma-treated cells were normalized and all results were presented as a percentage of secretion.

### 2.3 Immunofluorescence

HUVECs were treated with control or COVID-19 plasma for 24 hours prior to fixing with 3% PFA. Cells were permeabilized, blocked, and stained with mouse anti–human VWF (kindly provided by Professor Jan Voorberg, Amsterdam, Netherlands) and rabbit anti–human angiopoietin-2. After incubation with secondary antibodies (donkey anti-mouse 488 and donkey anti-rabbit 568) and DAPI for nucleus staining, confocal microscope images (Zeiss LSM 710 NLO) were analyzed with ImageJ.

### 2.4 Tube formation assay

HUVECs were trypsinized, resuspended in RM, control, or COVID-19 plasma, and then seeded onto ibidi μ-Slides Angiogenesis precoated with Matrigel. After 6 hours, images were collected and analyzed with an Angiogenesis Analyzer plugin for ImageJ.

Statistical analyses were performed using the Kruskal-Wallis test for multiple comparisons and the Spearman rank correlation in GraphPad Prism 9.0 (GraphPad Software) with a *P* value of <.05 considered statistically significant.

## 3 Results and Discussion

**Figure 1:**
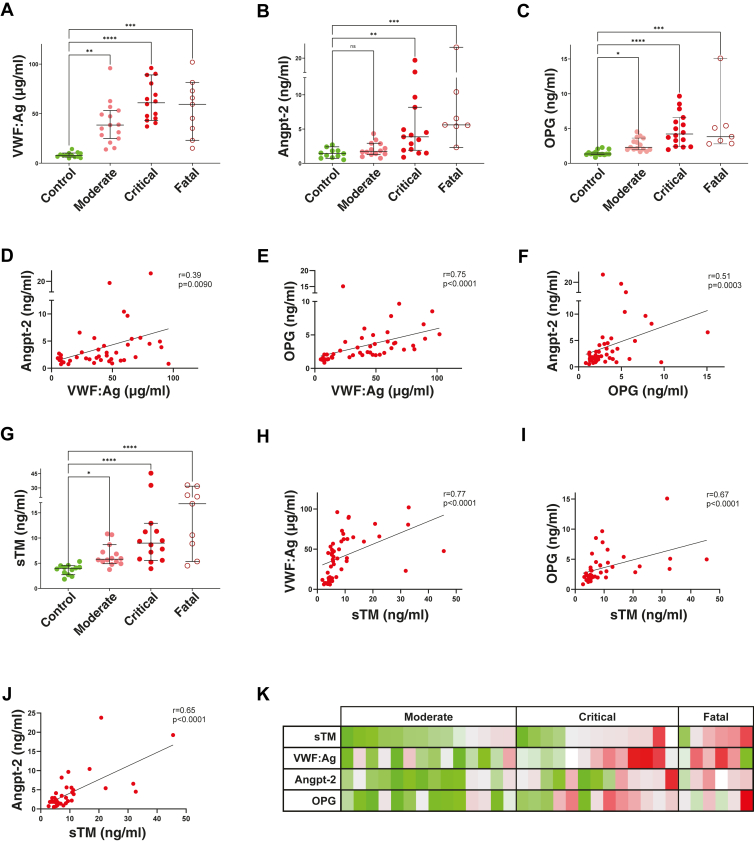
Comparison of WPB biomarker plasma levels in patients with acute COVID-19 (n = 39) compared to healthy controls (n = 15) are shown, including (A) VWF:Ag, (B) Angpt-2, and (C) OPG levels, respectively. All experiments were performed in triplicate and data are represented as a median with 95% CI unless stated otherwise. Comparison between groups were assessed by the Kruskal-Wallis test (ns, not significant: ∗*P* < .05; ∗∗*P* < .01; ∗∗∗*P* < .001; ∗∗∗∗*P* < .0001). (D-F) Correlations between plasma levels of various WPB components: (D) VWF:Ag vs Angpt-2, (E) VWF:Ag vs OPG, and (F) OPG vs Angpt-2. Correlations were evaluated using the Spearman rank correlation test. (G) Plasma soluble thrombomodulin (sTM) levels in patients with acute COVID-19 compared to controls. (H-J) Correlations between plasma sTM levels and WPB biomarkers: (H) sTM vs VWF:Ag, (I) sTM vs OPG, and (J) sTM vs Angpt-2. (K) Heatmap visualization indicating that sTM and WPB biomarker levels were detected in each subject (columns) for each protein (rows) categorized based on the COVID-19 severity. Levels were annotated from green (low) to red (high). Angpt-2, Angiopoietin-2; OPG, Osteoprotegerin; sTM, soluble thrombomodulin; VWF:Ag, von Willebrand factor antigen; WPB, Weibel-Palade bodies.

A total of 39 patients (14 with moderate, 16 with critical, and 9 with fatal COVID-19) were studied. The patients had a median age of 63 (IQR: 53-69) years. The majority were males (29/39, 74%) with underlying comorbidities (36/39, 92%). The median co-morbidity count was 3 (IQR: 2-3). Overall, the median length of hospital stay was 20 (IQR: 13.5-36) days, and was significantly longer in critical compared with moderate COVID-19 cases (median: 20 vs 15 days; *P* = .006). The median length of ICU stay was 20 (IQR: 9.5-39) days (Table). Consistent with previous reports, [7,8] plasma VWF levels were significantly elevated in patients with COVID-19 compared to healthy controls (Figure 1A). Furthermore, VWF:Ag levels were higher in patients with critical or fatal COVID-19 compared with moderate disease (median levels: 59.5 μg/mL in fatal and 61.0 μg/mL in critical COVID-19 vs 38.5 μg/mL in moderate COVID-19) (Figure 1A). Angpt-2 is another EC glycoprotein stored in WPB reported in recent studies that binds directly to the A1 domain of VWF. [14] With respect to severe COVID-19, Angpt-2 is a ligand of the Tie-2 receptor and has been shown to destabilize ECs, with effects on EC survival, vascular stability, endothelial permeability, and angiogenesis. [15] Similar to VWF and previous reports, [16,17] plasma Angpt-2 levels were significantly increased in patients with critical and fatal COVID-19 (median levels: 5.60 ng/mL in fatal and 3.87 ng/mL in critical COVID-19 vs 1.42 ng/mL in healthy controls) (Figure 1B). Interestingly, in contrast to VWF, plasma Angpt-2 levels were not significantly elevated in patients with moderate COVID-19 compared to controls (Figure 1B).

Osteoprotegerin is an EC protein that binds to VWF and thus is also recruited into WPBs. Osteoprotegerin has an established role in regulating bone remodeling by serving as a decoy receptor for RANKL. [18] Recent studies have also identified novel vascular functions for osteoprotegerin. For example, osteoprotegerin causes enhanced adhesion molecule expression on ECs and also influences EC apoptosis. [19] We observed that plasma osteoprotegerin levels were markedly elevated in patients with acute COVID-19 (Figure 1C). Consistent with the VWF data, osteoprotegerin levels were significantly elevated in all 3 subgroups of COVID-19 patients (median levels: 3.82 ng/mL in fatal COVID-19, 4.22 ng/mL in critical COVID-19, and 2.30 ng/mL in moderate COVID-19 respectively) (Figure 1C). To our knowledge, this represents the first report of significantly elevated plasma osteoprotegerin levels in severe COVID-19. However, markedly increased osteoprotegerin levels have also recently been described in children with cerebral malaria, in which they correlated with disease severity. [20] Importantly, cerebral malaria is also characterized by fulminant endotheliopathy that ultimately leads to microvascular occlusion. Consistent with the hypothesis that acute COVID-19 is associated with marked EC activation and secondary WPB exocytosis, significant correlations were observed between plasma VWF:Ag, Angpt-2, and osteoprotegerin levels, respectively (Figure 1D–F). Of note, although all 3 WPB biomarkers were markedly elevated in patients with critical or fatal COVID-19, only VWF:Ag and osteoprotegerin were significantly increased in cases with moderate COVID-19. These findings are interesting given that emerging data suggest that different subpopulations of WPB that differ in terms of their stored cargo proteins may exist within ECs. [21] In addition, heterogeneity in WPB distribution has also been described for lung microvascular EC. [22,23] Moreover, WPB secretion may differ depending upon whether it is triggered by increases in intracellular c-AMP and/or Ca^2+^, respectively. For example, osteoprotegerin secretion predominantly occurs in response to increased intracellular Ca^2+^ levels. [24] Collectively, our data support the hypothesis that EC activation and WPB secretion in severe COVID-19 are likely multifactorial in nature, and further suggest that the pathobiological mechanisms underlying this endotheliopathy may vary during the course of the illness.

Thrombomodulin (TM) is an EC surface receptor that promotes thrombin-induced activation of zymogen protein C. In addition, recent studies have identified an exciting new role for TM in maintaining normal EC quiescence. [25] Consequently, TM downregulation in EC was associated with loss of EC quiescence and significantly increased VWF biosynthesis and secretion. [25] In the context of severe COVID-19 endotheliopathy, this role for TM is interesting as increased soluble TM levels (sTM shed from EC surfaces) have been associated with poor clinical outcomes in patients with COVID-19. [4] Consistent with these data, plasma sTM levels were significantly elevated in all our COVID-19 patient subgroups compared to the control group (median levels: 16.78 ng/mL in fatal COVID-19; 8.99 ng/mL in critical COVID-19; and 5.80 ng/mL in moderate COVID-19 cases compared with 3.96 ng/mL in healthy controls) (Figure 1G). In addition, plasma sTM levels correlated significantly with VWF:Ag, osteoprotegerin, and Angpt-2, respectively (Figures 1H–K). Together, these findings suggest that loss of TM in acute COVID-19 accompanies EC activation, which in turn triggers enhanced WPB exocytosis.

**Figure 2:**
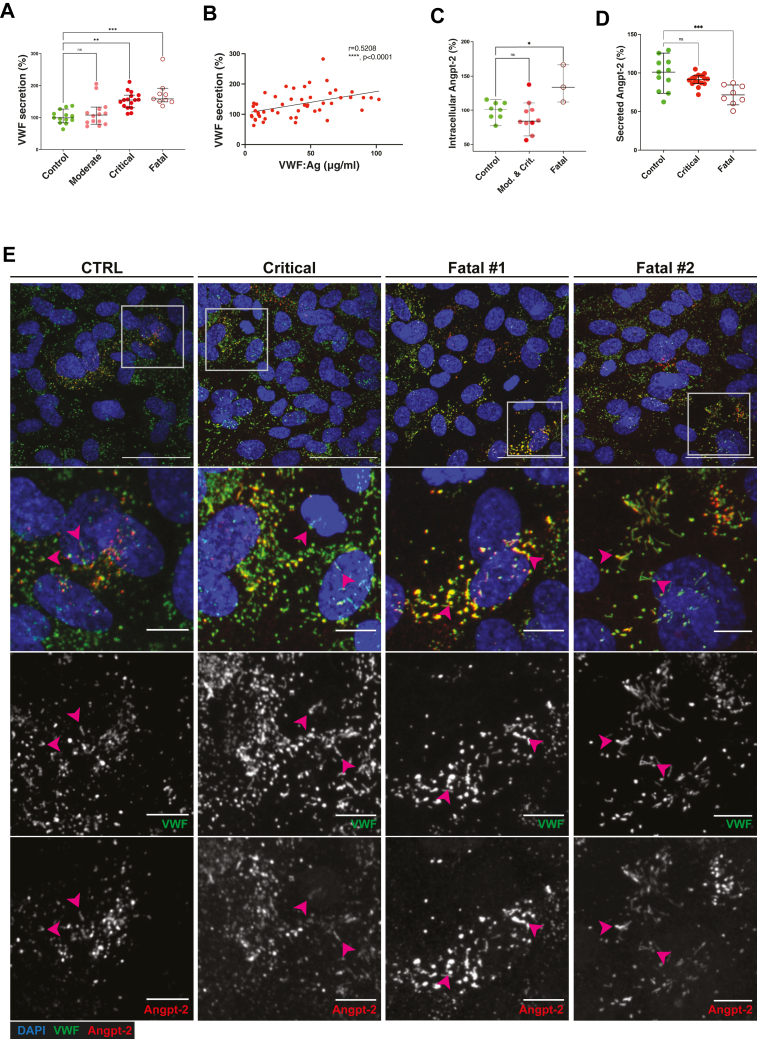
(A) Effects of COVID-19 plasma incubation on EC biology *ex vivo*. VWF secretion (%) from HUVECs after treatment with either healthy control plasma or acute COVID-19 plasma (moderate, critical, or fatal, respectively). All experiments were performed in triplicate and data are represented as a median with 95% CI unless stated otherwise. Comparison between groups were assessed by the Kruskal-Wallis test (ns, not significant: ∗*P* < .05; ∗∗*P* < .01; ∗∗∗*P* < .001). (B) Correlation between endogenous plasma VWF:Ag levels and *ex vivo* VWF secretion from HUVECs incubated with acute COVID-19 plasma from the same patient. (C) Secreted Angpt-2 after HUVEC stimulation with control, critical, and fatal COVID-19 plasma. Experiments were performed in triplicates. Comparisons between groups were assessed by the one-way ANOVA test (ns, not significant: ∗∗∗*P* < .001). (E) Representative immunofluorescent images of ECs after plasma treatment (DAPI [blue] stains the nucleus; VWF is in green, and Angpt-2 is in red [co-localization is shown in yellow]). VWF and Angpt-2 channels alone are also shown in gray scale (scale bar is set at 50 μm for the overview images and 10 μm for the zoomed-in regions; arrow heads point to WPBs positive for Angpt-2). (D) Intracellular Angpt-2 levels after incubation with control, moderate, and critical and fatal COVID-19 represented as median with 95% CI. Experiments were performed in duplicates. Comparison between groups was assessed by the one-way ANOVA test (ns, not significant: ∗*P* < .05). Angpt-2, Angiopoietin-2; Ctrl, control; DAPI, 4′,6-diamidino-2-phenylindole, nucleus staining; EC, endothelial cell; HUVEC, human umbilical vein endothelial cell; OPG, osteoprotegerin; sTM, soluble thrombomodulin; TM, thrombomodulin; VWF:Ag, von Willebrand factor antigen; WPB, Weibel-Palade bodies.

To investigate whether alterations in plasma in severe COVID-19 play a role in EC activation, human ECs were treated with COVID-19–or control-plasmas *ex vivo*. We observed a significant increase in VWF secretion when HUVECs were incubated with plasma from patients with critical or fatal COVID-19 (Figure 2A). Furthermore, *ex vivo*-induced VWF secretion correlated significantly with the endogenous plasma VWF:Ag levels observed in patients with acute COVID-19 (Figure 2B). In contrast, we found that incubation of plasma from moderate COVID-19 cases did not stimulate enhanced VWF secretion from HUVECs (Figure 2A). This is interesting because endogenous plasma VWF:Ag levels were significantly elevated in moderate COVID-19 (Figure 1A). These data suggest that mediators in plasma play an important role in promoting EC activation in the later stages of COVID-19, but that other mechanisms may be more important in early-stage endotheliopathy. As acute COVID-19 progresses, a positive feedback loop is established wherein EC activation leads to coagulation activation and proinflammatory changes in plasma, which in turn then exacerbate endotheliopathy. Incubation with fatal COVID-19 plasma resulted in a significant reduction in secreted levels of Angpt-2 from HUVECs (Figure 2C). To further investigate the effects of acute COVID-19 plasma on EC, immunofluorescent staining for VWF and Angpt-2 and intracellular Angpt-2 levels were assessed. A significant increase in intracellular Angpt-2 levels was observed in HUVECs incubated with fatal COVID-19 plasma compared to control, moderate, or critical COVID-19 plasma (Figure 2D). This finding is consistent with previous studies that reported a significant increase in *Angpt-2* transcription in HUVECs incubated with plasma from patients with severe COVID-19. [26] The majority of this Angpt-2 remained colocalized with VWF within WPBs (Figure 2E). Together, these Angpt-2 data are interesting as angiopoietins have been reported to bind TM and inhibit its anticoagulant function. [27] Thus, in severe COVID-19, multiple mechanisms are likely to contribute to a significant reduction in generation of anticoagulant and anti-inflammatory activated protein C generation on the EC surface.

**Figure 3:**
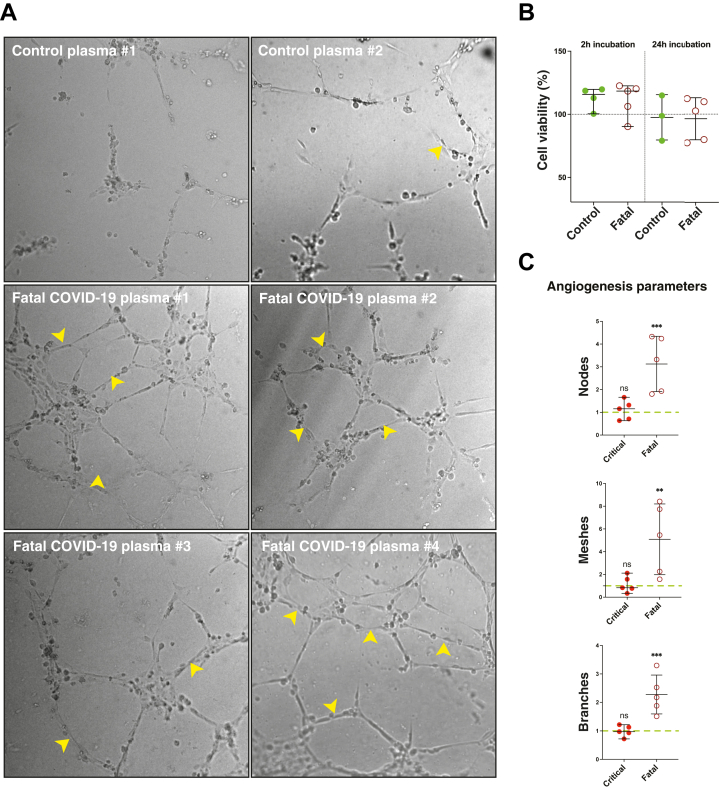
(A) Representative images of HUVEC tube formation after incubation with acute COVID-19 plasma compared to control plasma (arrowheads point to meshes). (B) Cell viability of HUVECs assessed by the WST-1 reagent after brief (2 hours) and prolonged (24 hours) incubation with control and fatal COVID-19 plasma. (C) Significantly increased nodes, meshes, and branches per field after HUVECs incubated with either fatal or critical COVID-19 plasma. All data shown have been normalized compared to control plasma-treated HUVECs. Comparisons between groups were assessed by one-way ANOVA test (ns, not significant: ∗∗*P* < .01; ∗∗∗*P* < .001). HUVEC, human umbilical vein endothelial cell; WST, Water-soluble Tetrazolium 1.

Autopsy studies have shown that acute COVID-19 is associated with a significant increase in angiogenesis. [1] In particular, intussusceptive angiogenesis and sprouting within the pulmonary vasculature were markedly elevated in COVID-19 compared to patients who had died from other types of viral infections. [1] Because VWF, Angpt-2, and osteoprotegerin have all been reported to influence angiogenesis, [15,18] we next studied whether COVID-19 plasma affected EC angiogenesis in a Matrigel-based tube formation assay. We observed a significant increase in all of the angiogenic parameters (nodes/meshes/branches per field) studied for HUVECs treated with plasma from patients with fatal COVID-19 compared to controls. In contrast, no significant changes in any of the angiogenesis parameters were observed in HUVECs treated with plasma from patients with critical COVID-19 (Figure 3). Altogether, these findings suggest that COVID-19 plasma not only has the potential to exacerbate endotheliopathy but also to significantly promote angiogenesis.

In conclusion, we provided evidence that multifactorial EC activation leads to marked WPB exocytosis during acute COVID-19. The increased levels of both sTM and WPB components (VWF, osteoprotegerin, and Angpt-2) correlate with the disease state, with more severely affected patients having the highest plasma levels. Based on these results, we propose that as COVID-19 illness develops, there is a progressive loss of TM from the EC surface. TM loss together with proinflammatory cytokines and other mechanisms contribute to multifactorial EC activation, WPB exocytosis, and increased Angpt-2 expression. The combination of increased Angpt-2 expression in ECs and WPB exocytosis then promoted a proangiogenic state. Additional studies will be required to define the biological mechanisms through which Angpt-2 and other factors contribute to abnormal angiogenesis in severe COVID-19, particularly in the setting of lung microvascular endothelial cells. In addition, additional research will be essential to characterize how these mechanisms vary during disease progression during SARS-CoV-2 infection.
